# Distribution of High-Risk Human Papillomavirus Genotypes among HIV-Negative Women with and without Cervical Intraepithelial Neoplasia in South Africa

**DOI:** 10.1371/journal.pone.0044332

**Published:** 2012-09-06

**Authors:** Alicia C. McDonald, Lynette Denny, Chunhui Wang, Wei-Yann Tsai, Thomas C. Wright, Louise Kuhn

**Affiliations:** 1 Department of Epidemiology, Mailman School of Public Health, Columbia University, New York, New York, United States of America; 2 Department of Obstetrics and Gynaecology, University of Cape Town, Cape Town, South Africa; 3 Department of Pathology, College of Physicians and Surgeons, Columbia University, New York, New York, United States of America; 4 Department of Biostatistics, Mailman School of Public Health, New York, New York, United States of America; 5 Gertrude H. Sergievsky Center, College of Physicians and Surgeons, Columbia University, New York, New York, United States of America; The Catalan Institute of Oncology (ICO), Spain

## Abstract

**Objective:**

Large studies describing the profile of high-risk Human papillomavirus (hrHPV) genotypes among women in sub-Saharan Africa are lacking. Here we describe the prevalence and distribution of hrHPV genotypes among HIV-negative women in South Africa, with and without cervical intraepithelial neoplasia (CIN).

**Methods:**

We report data on 8,050 HIV-negative women, aged 17–65 years, recruited into three sequential studies undertaken in Cape Town, South Africa. Women had no history of previous cervical cancer screening. Cervical samples were tested for hrHPV DNA using the Hybrid Capture 2 (HC2) assay and all positive samples were genotyped using a PCR-based assay (Line Blot). Women underwent colposcopy and biopsy/endocervical curettage to determine CIN status. The prevalence and distribution of specific hrHPV genotypes were examined by age and CIN status.

**Results:**

Overall, 20.7% (95% CI, 19.9–21.6%) of women were hrHPV-positive by HC2, with women with CIN having the highest rates of positivity. Prevalence decreased with increasing age among women without CIN; but, a bimodal age curve was observed among women with CIN. HPV 16 and 35 were the most common hrHPV genotypes in all age and CIN groups. HPV 45 became more frequent among older women with CIN grade 2 or 3 (CIN2,3). Younger women (17–29 years) had more multiple hrHPV genotypes overall and in each cervical disease group than older women (40–65 years).

**Conclusion:**

HPV 16, 35, and 45 were the leading contributors to CIN 2,3. The current HPV vaccines could significantly reduce HPV-related cervical disease; however, next generation vaccines that include HPV 35 and 45 would further reduce cervical disease in this population.

## Introduction

Human papillomavirus (HPV) is one of the most common sexually transmitted infections worldwide and most women are infected with one or more HPV genotypes at some point during their sexual lives. Persistent HPV infection caused by high-risk HPV (hrHPV) genotypes (16, 18, 31, 33, 35, 39, 45, 51, 52, 56, 58, 59, and 68) is associated with cervical pre-malignant lesions and cervical carcinoma, a cancer that is the second most common malignancy among women worldwide [1], [2], [3]. Globally, HPV 16 and 18 are the predominant hrHPV genotypes among women with invasive cervical cancer and cause approximately 50% and 20% of cervical cancers, respectively [4], [5]. Two highly effective prophylactic vaccines have been developed to prevent infection with HPV 16 and 18 [6], [7]. There are also several approved assays that have been used to improve cervical cancer screening by detecting hrHPV genotypes and determining the presence of a specific hrHPV genotype in women [3], [8]. HPV vaccination and cervical cancer screening with hrHPV testing in combination offer the potential of substantially reducing cervical cancer incidence in high-risk populations.

Although the prevalence of HPV infection is particularly high in women living in sub-Saharan Africa, studies have found that the proportion of high-risk infections attributable to HPV 16, the most common high-risk type, is less among women living in sub-Saharan Africa compared to women living in other regions of the globe [9], [10]. The lesser contribution of HPV 16 is somewhat surprising given that the incidence and mortality rates of invasive cervical cancer (31.7 and 22.9 per 100,000 women per year, age-standardized, respectively) in sub-Saharan Africa are among the highest in the world [2], [11], [12]. If HPV 16 contributes a lesser proportion of hrHPV types detected in cervical disease and cancer cases in sub-Saharan Africa compared to other geographical regions, HPV vaccination and screening programs that utilize HPV 16 and 18 genotyping assays to improve specificity would not have as great of an impact as in other regions where the proportion of disease due to HPV 16 is higher. Therefore, it is important to better describe the prevalence of specific hrHPV genotypes in sub-Saharan Africa women with and without cervical disease. A description of hrHPV genotypes will allow policy makers to identify the best strategies for reducing cervical cancer in this region.

Several meta-analyses have described the global distribution of HPV genotypes; however, sub-Saharan African populations contribute only relatively small amounts of data to these analyses [4], [9]. Moreover, HPV studies conducted in Africa have been heterogeneous in terms of age distribution, methods of ascertaining cervical disease, and the extent of HIV testing. As a result, age- and type-specific data for HIV-uninfected women of known cervical disease status are only imprecisely determined for any sub-Saharan African population. In this study, we described the prevalence and distribution of hrHPV genotypes, including multiple types, among HIV-negative women enrolled in three sequential cervical cancer screening studies undertaken in South Africa in which cervical disease status was rigorously determined using repeat colposcopy and biopsy [13], [14].

## Methods

### Study Population

This analysis is based on data from three sequential studies that recruited women from the same three clinical sites in the peri-urban community of Khayelitsha, outside Cape Town, South Africa. All three studies included women who were not pregnant at the time of enrollment, had never been screened or treated for cervical cancer, and had not undergone a hysterectomy.

#### Ethics statement

All women provided written informed consent and the protocols were approved by the Institutional Review Boards of Columbia University, New York and the University of Cape Town, South Africa. All clinical investigation was conducted according to the principles expressed in the Declaration of Helsinki.

In Cohort 1, 2,699 women (2,505 HIV-negative), aged 35–65 years, were enrolled between January 1998 and November 1999 into a study evaluating the performance of different tests for cervical cancer screening [14]. In Cohort 2, 6,553 women (5,708 HIV-negative), aged 35–65 years, were enrolled between June 2000 and December 2002 and were followed for 36 months in a trial examining the safety and efficacy of two screen-and-treat approaches for cervical cancer prevention [13]. For the analyses presented here, only women randomized to the control group (2,165; 1,881 HIV-negative) or to the screen-and-treat arm utilizing HPV testing (2,163; 1,874 HIV-negative) were included due to the availability of HPV typing data. In Cohort 3, 2,998 women (2,265 HIV-negative), aged 17–34 years, were enrolled in a study between July 2004 and June 2006 examining HPV prevalence among younger women. There were no duplicate women in the 3 cohorts to our knowledge. We further restricted our study population to women with definitive cervical disease status and excluded women with invasive cervical cancer due to the small number of cases (n = 23), resulting in a final sample size of 8,050 HIV-negative women (2,485 in Cohort 1, 3,353 in Cohort 2, and 2,212 in Cohort 3).

### Data Collection and Laboratory Procedures

In all three studies, a short risk factor questionnaire was conducted at baseline and blood was collected for HIV testing. A gynecologic examination was conducted in which cervical samples were obtained, including a sample collected from the exo- and endocervix using a plastic spatula and cytobrush and placed into liquid-based cytology medium (ThinPrep PreservCyte, Hologics, Marlborough, MA). Pap smears were evaluated at either the University of Cape Town Cytopathology Laboratory, Health Networks Laboratory, Allentown PA, or Columbia University, New York, NY and classified using the Bethesda System. Hybrid Capture 2 (HC2) DNA assay (Qiagen Corporation, Germantown, MD, USA) was used to test cervical samples for hrHPV DNA types 16, 18, 31, 33, 35, 39, 45, 51, 52, 56, 58, 59, and 68 [13]. High-risk HPV DNA positivity was based on a cut-off of relative light units (RLU)>1 times the positive control. Aliquots of the liquid-based cytology samples were stored at −30°C for future testing.

After the fieldwork for the studies were completed, stored cervical samples from all women who were HC2 positive were sought for determination of the specific hrHPV genotype present. Of 8,050 women in the three cohorts, 1,670 tested hrHPV DNA positive by HC2 and 1,642 (98.3%) could be located and tested to determine the specific high risk HPV genotype present.

For hrHPV genotyping, DNA was isolated from 200 ul of the liquid-based cytology specimen using a spin column (Qiagen Inc., Chatsworth, CA.). Purified DNA was analyzed for individual HPV genotypes using a prototype polymerase chain reaction-based (PCR) line blot assay (kindly provided by Dr. Janet Kornegay, Roche Molecular Diagnostics, Alameda, CA) that uses the PGMY09/11 consensus primers. If a hrHPV genotype was not identified using the prototype line blot assay, DNA was re-isolated and re-analyzed for individual HPV genotypes using the PCR-based, Linear Array HPV Typing Assay (Roche Molecular Diagnostics, Alameda, CA) [15].

**Table 1:** Characteristics of 8050 HIV-negative women recruited into three cervical cancer screening studies in Khayelitsha, South Africa.

	Cohort 1 (n = 2485)	Cohort 2 (n = 3353)	Cohort 3 (n = 2212)	P-value (Younger vs Older Cohorts)
**Age range**	35–65 years	35–65 years	17–34 years	
**Median Age ** ***(25–75*** ***percentile)***	41 years *(36–47 years)*	42 years *(38–48 years)*	26 years *(22–30 years)*	<0.0001
**N (%) < = 10 years of Education**	2243 (90.26%)	3075 (91.71%)	1223 (55.29%)	<0.0001
**N (%) Currently employed**	1042 (42.25%)	874 (26.07%)	603 (27.26%)	<0.0001
**N (%) Smoker**	128 (5.16%)	239 (7.13%)	65 (2.94%)	<0.0001
**N (%) Drink alcohol in last** **month**	167 (6.72%)	416 (12.41%)	328 (14.83%)	<0.0001
**N (%) Married**	1167 (46.96%)	1789 (53.36%)	823 (37.21%)	<0.0001
**Median age of 1^st^ sexual** **intercourse ** ***(range)***	17 years (10–30 years)	16 years (6–39 years)	17 years (7–28 years)	0.0853
**N (%) Ever treated for STD**	42 (1.70%)	93 (2.77%)	523 (23.64%)	<0.0001
**N (%) Ever used condoms**	187 (9.08%)	236 (8.30%)	1341 (60.62%)	<0.0001
**N (%) >2 live births**	1653 (66.63%)	2496 (74.44%)	191 (8.63%)	<0.0001
**N (%) Disease Status**				
WNL	2328 (93.7%)	3219 (96.0%)	2022 (91.4%)	<0.0001*
CIN 1	79 (3.2%)	52 (1.6%)	130 (5.9%)	
CIN 2	41 (1.6%)	51 (1.5%)	40 (1.8%)	
CIN 3	37 (1.5%)	31 (0.9%)	20 (0.9%)	
**N (%) HC2 HPV** **DNA Positive**	429 (17.26%)	572 (17.06%)	669 (30.24%)	<0.0001

Note: * = p-value of trend, STD = sexually transmitted disease, WNL = within normal limits, CIN 1 = cervical intraepithelial neoplasia grade 1, CIN 2 = cervical intraepithelial neoplasia grade 2, CIN 3 = cervical intraepithelial neoplasia grade 3, HPV = Human papillomavirus, HC2 = Hybrid capture 2, Older cohort (aged 35–65 years) = Cohorts 1 and 2 combined, Younger cohort (aged 17–34 years) = Cohort 3, Pearson’s chi-square test and Student’s T-test were used to examine differences between the older and younger cohorts.

### Determination of Disease Status

To meet the objectives of each study, slightly different protocols were followed to determine disease status (within normal limits [WNL], cervical intraepithelial neoplasia [CIN] grade 1 [CIN1], CIN grade 2 [CIN2], CIN grade 3 [CIN3]) in each cohort. In Cohort 1, all women who had positive results on one or more of four independent screening tests were referred for colposcopy 2–6 days after the enrollment visit. The four screening tests were HPV DNA testing using HC2 (RLU>1x were referred), visual inspection with acetic acid, cytology (ASCUS and above were referred), and expert cervicography [14]. Approximately half of the participants had one or more of the four screening tests classified as positive and underwent colposcopy. In Cohort 2, colposcopy was performed on all women at 6 and 12 months after enrollment [13]. Samples for HPV DNA testing were collected at the time of enrollment. In Cohort 3, all women underwent colposcopy at their enrollment examination. Women not found to have biopsy-confirmed CIN2 or greater at the initial colposcopy who were HC2 positive, had cytology results of ≥ASCUS, or who had biopsy-confirmed CIN1 lesions underwent a second colposcopy 12 weeks after enrollment. Thus, for both Cohorts 2 and 3, all subjects underwent at least one or more colposcopy examinations. Due to the fact that no cervical disease was diagnosed in Cohort 2 or 3 among women who had both negative HPV and cytology results, we can confirm that minimal verification bias exists in Cohort 1. In all studies, colposcopy was conducted by clinicians specifically trained in colposcopy and according to a standard protocol. All abnormal areas were biopsied and endocervical curettage specimens were collected. Biopsy and endocervical curettage specimens were evaluated by two pathologists at Columbia University. Inconsistent diagnoses were adjudicated in a microscopic conference and the final disease status is based on the highest grade adjudicated pathology diagnosis.

**Figure 1:**
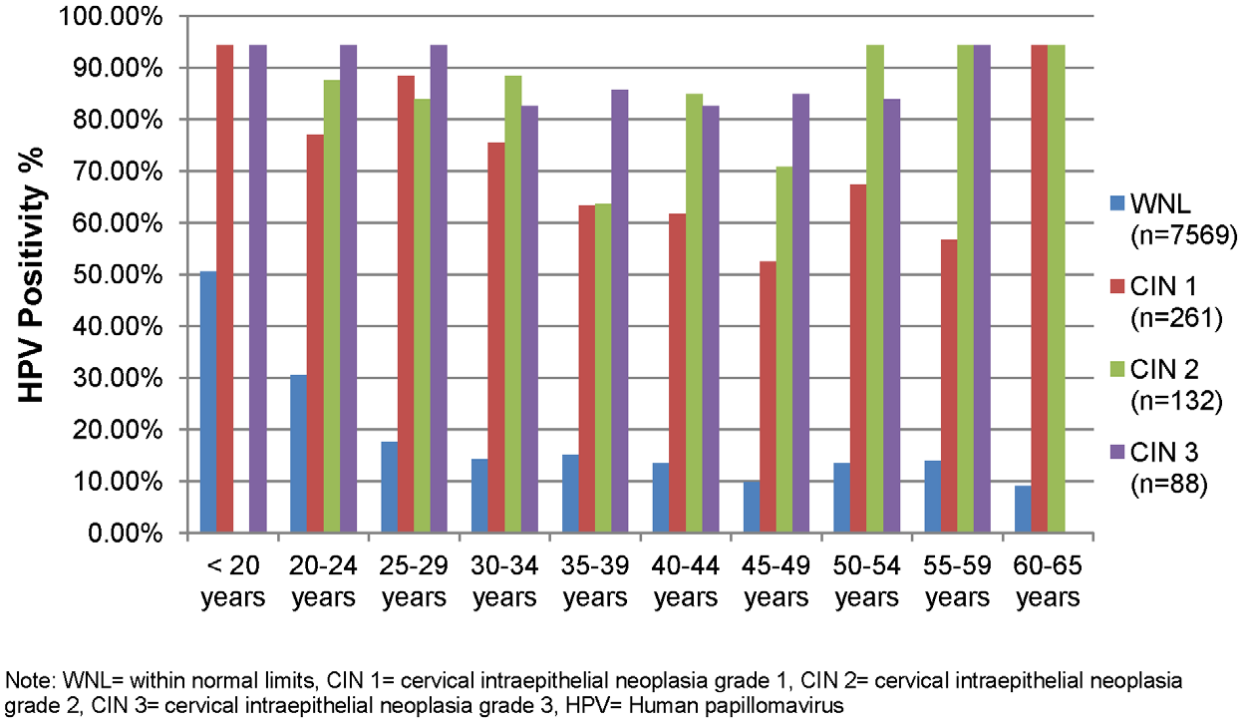
Age-specific high-risk HPV DNA prevalence (Hybrid Capture 2) by cervical disease status among 8,050 HIV-negative women in Khayelitsha, South Africa.

### Data Analysis

Two parameters were calculated to describe the epidemiology of hrHPV genotypes: (1) distribution and (2) prevalence. Distribution refers to the proportion of the hrHPV infection burden attributable to a specific genotype and was calculated in two ways: a) utilizing the total number of *women* with any hrHPV type as the denominator, and b) utilizing the total number of all hrHPV *infections* as the denominator. For genotype-specific prevalence calculations, we assumed that the HC2 assay was100% sensitive and specific in detecting hrHPV. Thus, type-specific HPV prevalence was calculated by multiplying the observed distribution of a specific type by the observed HPV prevalence determined by HC2.

**Figure 2:**
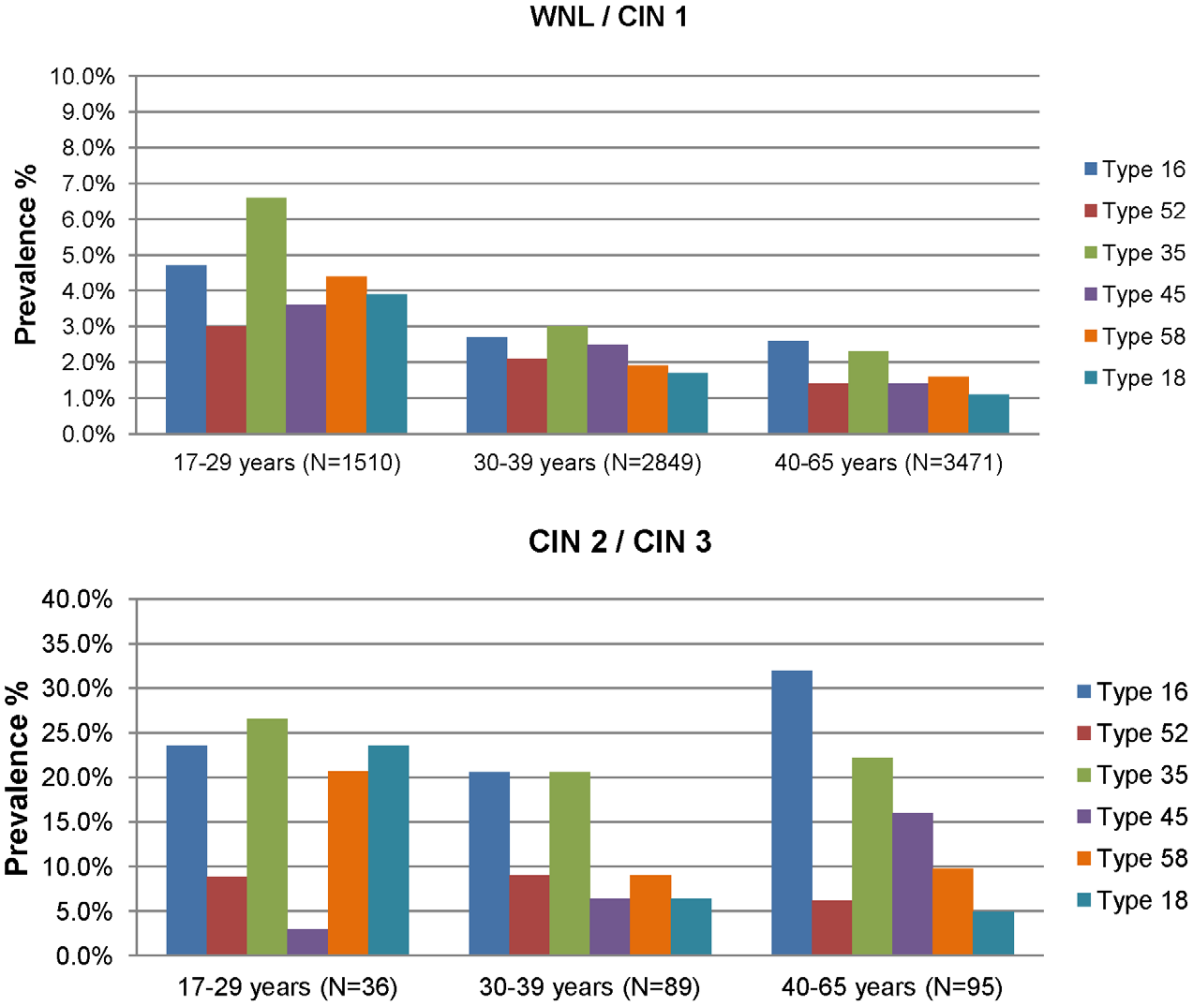
Age- and genotype-specific prevalence of the 6 most common high-risk HPV genotypes among 8,050 HIV-negative women.

HPV prevalence determined by HC2 positivity, the distribution and prevalence of specific hrHPV genotypes, and the frequency of multiple types was examined by cervical disease status and age group. Pearson’s chi-square test or Fisher’s exact test (when appropriate) were used to compare frequencies between groups in distribution and frequency of multiple hrHPV genotypes. For type-specific prevalence comparisons, the standard errors for the estimates were calculated using the delta method and compared to a normal distribution. Analysis was conducted using SAS statistical software (Cary, NC).

## Results

### Study Population

There were 8,050 HIV-negative women, aged 17–65 years, included in this analysis. Table 1 describes demographic, behavioral, and clinical characteristics by study cohort. Women in the younger age cohort, aged 17–34 years, were more educated, less likely to be married, more likely to be treated for a sexually transmitted disease, and more likely to test HC2 HPV DNA positive in comparison to women in the older age cohorts, aged 35–65 years (p<0.0001). In addition, women in the younger age cohort were more likely to be diagnosed with CIN1 than women in the older age cohorts. Women in the older age cohorts had more live births and used condoms less than women in the younger cohort (p<0.0001).

**Table 2:** Distribution of specific high-risk HPV genotypes among 1,239 HIV-negative women with a high-risk genotype detected by PCR.

	WNL	CIN 1	CIN 2	CIN 3	P-value(WNL/CIN 1 vs CIN 2,3)
**# Women with a HR Types**	902	172	93	72	–
**# of isolates**	1164	247	126	100	–
**N (%) Genotype**					
** Type 16**	146 (16.19)	23 (13.37)	21 (22.58)	29 (40.28)	**<0.0001**
** Type 35**	151 (16.74)	41 (23.84)	29 (31.18)	14 (19.44)	**0.0125**
** Type 33**	67 (7.43)	15 (8.72)	12 (12.90)	9 (12.50)	**0.0274**
** Type 45**	106 (11.75)	19 (11.05)	7 (7.53)	12 (16.67)	0.9632
** Type 58**	115 (12.75)	14 (8.14)	13 (13.98)	9 (12.50)	0.6288
** Type 31**	72 (7.98)	17 (9.88)	8 (8.60)	9 (12.50)	0.3886
** Type 18**	85 (9.42)	22 (12.79)	9 (9.68)	8 (11.11)	0.8921
** Type 52**	90 (9.98)	21 (12.21)	12 (12.90)	3 (4.17)	0.6225
** Type 39**	51 (5.65)	7 (4.07)	1 (1.08)	2 (2.78)	**0.0516**
** Type 56**	56 (6.21)	15 (8.72)	3 (3.23)	1 (1.39)	**0.0346**
** Type 59**	65 (7.21)	14 (8.14)	2 (2.15)	1 (1.39)	**0.0041**
** Type 51**	75 (8.31)	23 (13.37)	5 (5.38)	1 (1.39)	**0.0179**
** Type 68**	85 (9.42)	16 (9.30)	4 (4.30)	2 (2.78)	**0.0141**
** Types 16/18**	229 (25.39)	43 (25.00)	29 (31.18)	35 (48.61)	**0.0003**

Note: WNL = within normal limits, CIN 1 = cervical intraepithelial neoplasia grade 1, CIN 2 = cervical intraepithelial neoplasia grade 2, CIN 3 = cervical intraepithelial neoplasia grade 3, HPV = Human papillomavirus, HC2 = Hybrid capture 2, HR = high-risk, 16/18 = HPV 16 and/or HPV 18, PCR = Polymerase chain reaction.

### Hybrid Capture 2 (HC2) HPV DNA Positivity

Overall, HC2 HPV DNA positivity was 20.7% (95% CI, 19.9–21.6%), with women in the younger cohort having the highest prevalence (30.2%, 95% CI, 28.3–32.2%) in comparison to women in the older cohorts (17.1%, 95% CI, 16.0–18.4%) (Table1)**.** Younger age was strongly associated with higher rates of HC2 HPV DNA positivity among women with WNL, with a positivity of 53.7% in women <20 years of age. HC2 HPV DNA positivity among women with WNL declined and stabilized around 15% by age 30 years; and, there was no clear evidence of a subsequent rise in older women (Figure 1). For women with cervical disease, HC2 HPV DNA positivity was high regardless of age with a suggestion of a U-shaped curved with the lowest rates of HPV DNA in the 30–49 year age group and higher rates in younger and older age categories (Figure 1).

**Figure 3:**
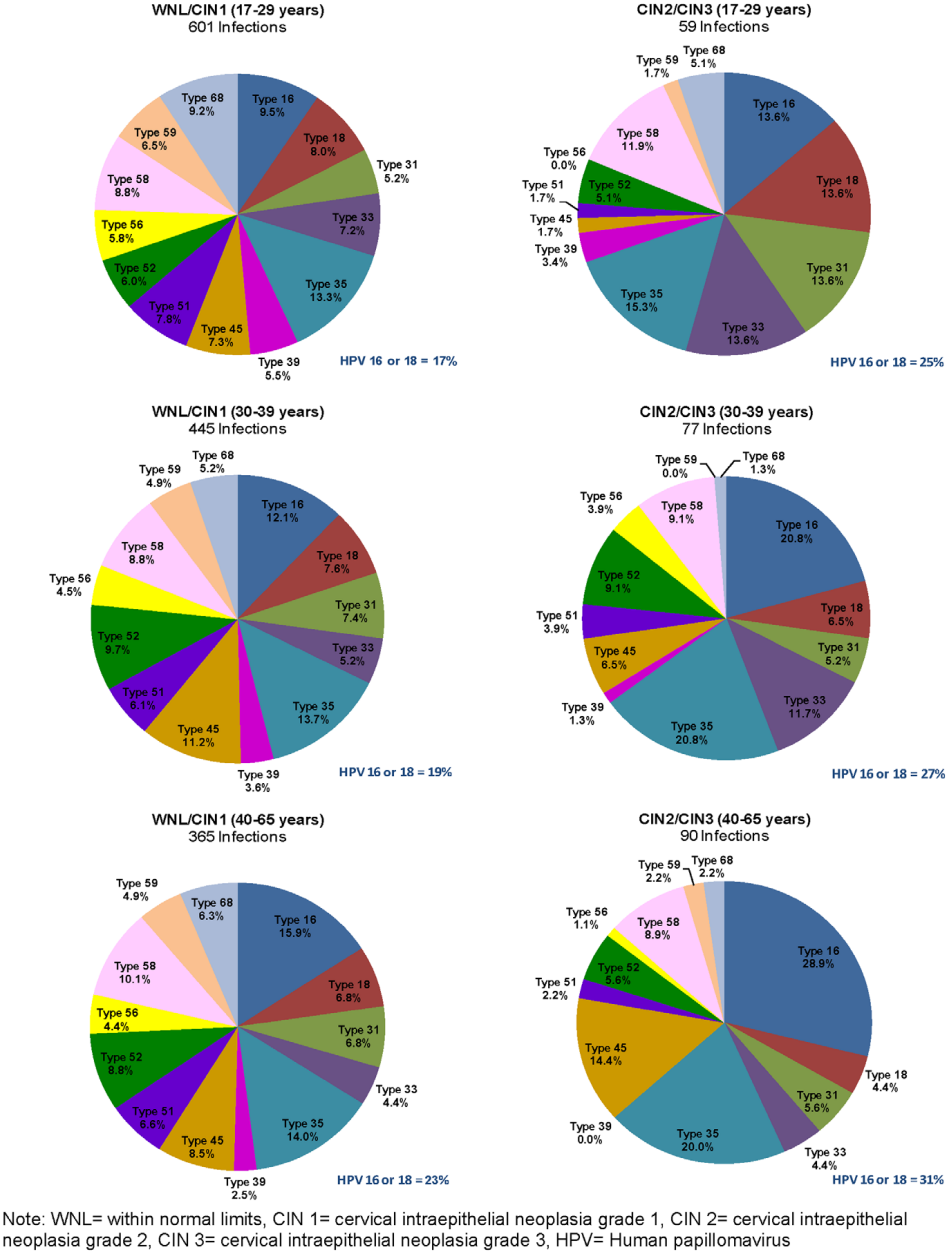
Distribution of high-risk HPV genotypes among 1,637 high-risk HPV infections in 1,239 women (i.e. a woman may have more than one type) by disease status and age.

### Genotype-specific Prevalence

Of 1,670 women who tested HC2 HPV DNA positive, samples from 1,642 (98.3%) could be located and tested to determine the specific high risk HPV genotype present. PCR identified one or more hrHPV genotype in 71.5% (902/1261) of women with WNL, 86.9% (172/198) of women with CIN1, 90.3% (93/103) of women with CIN2, and 90% (72/80) of women with CIN3. The most common hrHPV genotypes in descending order were HPV 35, 16, 58, 45, 52 and 18. The three most common HPV genotypes in descending order by disease status were in women with WNL: HPV 35, HPV 16, and HPV 58; in women with CIN1: HPV 35, HPV 16 & 51 (tied), and HPV 18, in women with CIN2: HPV 35, HPV 16, and HPV 58; and in women with CIN 3: HPV 16, HPV 35, and HPV 45. A high-risk HPV genotype was identified in 18 of the 23 invasive cervical cancer cases. In these 18 cases the most common genotypes were as follows: HPV 16 (56%), HPV 18 (22%), HPV 45 & 58 (tied- 11%), and HPV 52 & 68 (tied –6%); none had HPV 35 detected.

**Table 3:** Multiple high-risk types among 1,239 HIV-negative women with a high-risk HPV genotype by age and disease status.

Disease Status	Age	N with at least1 HR type	Median *(range)*	N with 1 Type (%)	N with 2+ Types (%)	P-value
**All**	17–29 years(reference group)	435	1 *(1–6)*	281 (64.60%)	154 (35.40%)	–
	30–39 years	420	1 *(1–4)*	337 (80.24%)	83 (19.76%)	<0.0001
	40–65 years	384	1 *(1–4)*	320 (83.33%)	64 (16.67%)	<0.0001
**WNL/CIN 1**	17–29 years	403	1 *(1–6)*	264 (65.51%)	139 (34.49%)	–
	30–39 years	358	1 *(1–4)*	288 (80.45%)	70 (19.55%)	<0.0001
	40–65 years	313	1 *(1–3)*	264 (84.35%)	49 (15.65%)	<0.0001
**CIN 2/CIN 3**	17–29 years	32	1 *(1–6)*	17 (53.13%)	15 (46.88%)	–
	30–39 years	62	1 *(1–3)*	49 (79.03%)	13 (20.97%)	0.0093
	40–65 years	71	1 *(1–4)*	56 (78.87%)	15 (21.13%)	0.0078
**Total Study Population**		**1239**	**1 ** ***(1–6)***	**939 (75.71%)**	**301 (24.29%)**	–

Note: WNL = within normal limits, CIN 1 = cervical intraepithelial neoplasia grade 1, CIN 2 = cervical intraepithelial neoplasia grade 2, CIN 3 = cervical intraepithelial neoplasia grade 3, HPV = Human papillomavirus.

Among women with WNL or CIN1 (WNL/CIN1), those who were younger (17–29 years) had a significantly higher prevalence of 5/6 most common hrHPV genotypes than older women. In contrast among women with CIN2 or CIN3 (CIN 2,3), younger women only had a higher prevalence of HPV 18 and 58 than older women. Other common types were either similar across age groups or tended towards an increased prevalence with older age (HPV16 and HPV45) (Figure 2).

Women with CIN 2,3 had a significantly higher prevalence of the 6 common hrHPV genotypes than women with WNL/CIN 1 (p<0.05) in all age strata. HPV 45 and HPV 52 were the exception among younger women (17–29 years) where there was no significant difference in the prevalence of these types by disease status (Table S1).

### Distribution of Specific hrHPV Genotypes

Table 2 displays the distribution of genotypes among women with at least one identified hrHPV genotype by disease status. HPV 35 and 16 were the most common hrHPV genotypes among women in every disease level. The proportions of hrHPV infections attributable to HPV 16, 35, and 33 were significantly higher in women with CIN 2,3 than in women with WNL/CIN 1 (p<0.03). In contrast, the proportions of hrHPV infections attributable to HPV 56, 59, 51, and 68 were significantly lower in women with CIN 2,3 than in women with WNL/CIN 1 (p<0.04). Considering the specific contribution of HPV types 16 and/or 18 (HPV 16/18), 27.1% of women had one or both of these hrHPV types. HPV 16/18 contributed to 48.6% of hrHPV infections among women with CIN 3, 31.2% among women with CIN 2, 25.0% among women with CIN 1, and 25.4% among women with WNL (Table 2).

Figure 3 shows the age- and disease-specific distribution of genotypes using all hrHPV infections as the denominator. HPV 35 and 16 remained the most common hrHPV genotypes in all age and disease groups. HPV 16 was more common than HPV 35 in older women (40–65 years) with or without cervical disease. In women with WNL/CIN 1, HPV 16 showed a significant trend towards increasing frequency with increasing age (p-_trend_ = 0.01). In women with CIN 2,3, HPV 45 showed a significant trend towards increasing frequency with increasing age (p_trend_ = 0.02). Only HPV 68 showed a significant decline in frequency with increasing age in women with WNL/CIN 1 (p_trend_ = 0.04). Considering the contribution of HPV16/18 to all hrHPV infections, HPV16/18 made up 35.0%, 23.0%, 17.4%, and 19.7% in women with CIN 3, CIN 2, CIN 1 and WNL, respectively.

### Multiple hrHPV Genotypes

Table 3 shows the frequency of multiple hrHPV genotypes by age and cervical disease status among women with at least one hrHPV genotype detected. Among these 1,239 women, 24% had multiple hrHPV genotypes detected, with some women having as many as 6 different hrHPV genotypes. The most common hrHPV genotypes among women with multiple types were the following in descending order: HPV 35, HPV 16, and HPV 58. Out of the 301 women with multiple HPV genotypes, co-infection with HPV 35 and HPV 16 was the most common followed by co-infection with HPV 35 and HPV 58. Younger women (17–29 years) were more likely to have multiple hrHPV genotypes than older women (30–65 years) overall (p<0.0001) and within each cervical disease group (p<0.01). Cervical disease status was unrelated to multiple hrHPV types, controlling for age.

## Discussion

Our study provides a comprehensive description of the profile of hrHPV infections among a large cohort of HIV-negative women in South Africa. We found that hrHPV infection is common among this population, with HPV 16 and 35 being the most common high-risk genotypes. Younger women had the highest burden of hrHPV infections and were more likely to be infected with multiple high-risk genotypes than older women. Interestingly, the proportion of cervical disease attributable to different HPV genotypes differed across age groups. Specifically, HPV 35, HPV 16, and HPV 45 made a relatively larger contribution to advanced cervical disease in older relative to younger women.

Overall the observed prevalence of hrHPV in HIV-negative women was 20.7% in our study. Compared to other studies using the same assay, hrHPV prevalence in our study was higher than reported in developed countries like Spain (10.7%) [16] and the U.S. (14.3%) [17], but lower than reported in Denmark (22.8%) [18]. Relative to studies conducted in Sub-Saharan Africa, the hrHPV prevalence in our study population fell between the prevalence of 10.2% reported among HIV-negative Ugandan women, aged 15–59 years [19] and 27.6% among HIV-negative Zimbabwean women, aged 25–55 years [20]. Age differences across the studies make comparisons difficult but differences across populations may be due to sexual behavior. Our finding of higher prevalence among younger women is consistent with most other studies [19], [20]. A possible explanation could be that younger women are more sexually-active, and therefore exposed to more hrHPV types than older women. The decrease of hrHPV prevalence with increasing age could also reflect clearance of hrHPV infections over time [21], [22].

As expected, the prevalence of hrHPV, all high-risk genotypes collectively and specific high-risk genotypes separately, was higher among women with CIN 2,3 than among women with WNL/CIN1. The prevalence of hrHPV DNA among women with advanced disease was >80%, similar to that reported from many studies and meta-analyses of women in Africa and other parts of the world [4]. Among women with CIN 2,3, a slight bi-modal curve of hrHPV prevalence was observed across age groups: the first peak among younger women, aged <20 years, and a second peak among older women. The slight reduction in hrHPV prevalence observed among middle-aged women with CIN2,3 may be a result of difficulties for the pathologist to discriminate between true CIN 2,3 and its histological mimics. Previously we have shown that many of the hrHPV negative CIN 2,3 lesions included in this analysis stain negatively for p16 and therefore appear to be histological mimics of CIN 2,3 [23]. Moreover, a recent study that found an apparent reduction in the prevalence of hrHPV in CIN 2,3 lesions with increasing age documented that many of the hrHPV negative lesions diagnosed as CIN 2,3 were also negative for p16 [24]. This interpretation is further strengthened by the finding that in the current study the bi-modal curve was difficult to discern in women with CIN 3 which is a more robust histological diagnosis than is CIN 2. Other possible explanations for the second peak of positivity among older women, which has been reported in previous studies [25], [26], may include changes in sexual behavior, a cohort effect, or HPV reactivation as a result of age-related declines in immune function or hormonal levels [27].

In this large South African population, HPV 35 and 16 were the two most common hrHPV genotypes, regardless of cervical disease status. Other studies conducted in Sub-Saharan Africa, including in South Africa [28], [29], Kenya [30], and Nigeria [31] also showed that HPV 35 was equal to or more common than HPV 16 among women with or without cervical disease. However, for the majority of these studies, HIV status was not known [28], [29], [30], [31]. In addition, we found that women with multiple high-risk genotypes were more likely to be infected with HPV 35 than any other high-risk type. Globally, HPV 35 has not been reported as the most common high-risk genotype among women with or without cervical disease [4], [9], [10], [27]; and, current HPV vaccines do not cover or produce significant cross-protection for this high-risk type [32], [33]. Therefore, women who are given the current vaccines are not protected against HPV 35, a high-risk type that is prevalent in Sub-Saharan Africa and is associated with cervical disease. We did not observe HPV 35 among the small number of invasive cancer cases were are able to test. The role of HPV35 in invasive cervical cancer among women in this region needs further investigation.

The prevalence of HPV 16 and 45 increased with age among women with CIN 2,3; and, these two types constituted a greater proportion of the high-risk infections in older women. The greater proportion of CIN 2,3 lesions associated with HPV 16 with increasing age observed in this South African population contrasts with the reduction in HPV 16 infections in CIN 2,3 lesions with increasing age that was recently reported from North America [24]. The reasons for these differential age interactions with HPV 16 are unclear but may relate to the fact that the South African women had not been previously screened whereas the North American women were a well-screened cohort. Other studies have shown that HPV 16-associated lesions are larger and presumably easier to detect through screening than lesions associated with other hrHPV genotypes [34]. For HPV 35, neither the prevalence nor the proportional contribution of this high-risk type differed by age among women with more advanced disease. Younger women, aged 17–29 years, had a wider variety of high-risk HPV genotypes, including HPV 35, and an equal distribution of HPV 33, HPV 31, HPV 18, and HPV 16, which may be due to increased sexual exposure to more hrHPV types than older women.

HPV 16 and/or 18, the high-risk types included in the licensed vaccines and reported to be the most prevalent high-risk HPV types worldwide, accounted for high risk infections among 25.4% of women without disease but 48.6% of high risk infections among women with CIN 3. These statistics under-estimate the value of the current vaccines as the contribution of HPV 16 and 18 is greater when analysis is restricted to cervical cancer cases. HPV 16 and 18 are highly associated with invasive cervical cancer, comprising together >70% of HPV types detected in this disease group, worldwide [4], [5], [35]. A significant limitation of our study is that only a small number of invasive cervical cancer cases were identified during screening limiting our capacity to comment on the hrHPV genotype distributions in this group. Meta-analyses have shown Africa as the only geographical area in which HPV 35 was among the top 5 most common hrHPV genotypes in women with invasive cervical cancer [4], [5]; the reason for this higher prevalence in Africa compared to other regions is not known but may relate to host genetic differences. Our results suggest that next generation vaccines that include HPV 35 could have significant impact on the decrease of cervical diseases in Sub-Saharan Africa.

We observed that younger women (17–29 years) were more likely to have multiple hrHPV genotypes compared to older women (≥30 years), regardless of cervical disease status. This observation is consistent with other studies that have examined multiple high-risk genotypes among younger versus older women [25], [36]. This higher frequency of multiple hrHPV genotypes among younger women may be due sexual activity and larger numbers of partners. Some studies have shown multiple HPV genotypes to be associated with HPV persistence [22], [37] which suggesting that they could contribute to the development of cervical neoplasia. However, in our study, multiple hrHPV infections were not associated with cervical disease after controlling for age.

There are limited data on the prevalence and distribution of specific high-risk HPV genotypes among HIV negative women in Sub-Saharan Africa populations by age and cervical disease. Our study population is large and HIV and cervical disease status were rigorously ascertained providing a comprehensive description of high-risk HPV genotypes among HIV negative women in South Africa. One limitation of the study was that some women were excluded in the final analysis as their cervical disease status could not be classified. However, these exclusions were limited (∼6% of the total cohort) and therefore unlikely to have affected the estimates. A second limitation was that HPV DNA negative women in Cohort 1 with a negative cytology did not have a colposcopy performed; as a result, cervical disease could have been missed in this group. However, our Cohort 2 women who were HPV negative and had a negative cytology did not have cervical disease detected by colposcopy which gives us confidence that cervical disease was unlikely to have been missed in Cohort 1. Another limitation is that PCR genotyping was only conducted on the samples that were HC2 positive. Since the HC2 assay is unlikely to be 100% specific to detect HPV DNA, some of the samples that were HC2 positive but had no detectable genotype may have been truly hrHPV negative. However, it is more likely that much of this discrepancy is explained by the less than perfect sensitivity of the genotyping assay and the fact that for genotyping we utilized archived samples that were several years old. We made the simplest possible assumptions in calculating the distribution and prevalence of specific types within the limitations of these current assays. Assay performance is unlikely to differ by genotype, [38] thus the higher than expected proportion with HPV35 is unlikely to be due to bias of the PCR assay used.

Our estimates may be biased but there is no evidence to suggest this to be the case. Measurement of HPV status and determination of cervical disease status at different time points, in particular, with Cohort 2, may be a limitation if HPV infection cleared before cervical disease ascertainment; as a result, we could have slightly over-estimated the hrHPV prevalence in women without disease. Finally, we had only a small number of invasive cervical cancer cases limiting our capacity to comment on the genotype distribution in this group.

### Conclusion

In this large cohort of HIV negative South African women spanning a wide age range from 18 to 65 years, HPV 16 and 35 were the most prevalent hrHPV genotypes, regardless of cervical disease status. Younger women had exceedingly high rates of hrHPV infection and could benefit from receiving the HPV vaccine prior to initiating sexual activity. We also observed interesting age relationships with HPV types 16 and 45 constituting a larger proportion of the infection burden in older relative to younger women with CIN 3. Although the currently-approved vaccines targeting HPV 16 and 18 could have a substantial impact on cervical disease in this population if initiated before their sexual debut, next generation vaccines that include other hrHPV genotypes, especially HPV 35 and HPV 45, will further reduce HPV infections in a population that is at high risk for advanced cervical disease and invasive cervical cancer.

## Supporting Information

Table S1 **Age- and genotype-specific prevalence of the 13 high risk HPV genotypes among 8,050 HIV-negative women providing more detail to** **Figure 2** **.**
(DOC)
