# Supplementary material for: Distribution of High-Risk Human Papillomavirus Genotypes among HIV-Negative Women with and without Cervical Intraepithelial Neoplasia in South Africa
Source: PLoS One. 2012 Sep 6;7(9):e44332. doi: 10.1371/journal.pone.0044332 (PMC3435398; doi:10.1371/journal.pone.0044332)
Supplement: Table S1 — Age- and genotype-specific prevalence of the 13 high risk HPV genotypes among 8,050 HIV-negative women providing more detail to Figure 2 . (DOC) [file pone.0044332.s001.doc]

Supplementary Table: Age- and genotype-specific prevalence of the 13 high risk HPV genotypes among 8,050 HIV-negative women providing more detail to Figure 2

|  | WNL & CIN 1 | | | P-values within WNL/CIN1 | | CIN 2 & CIN 3 | | | P-values within  CIN 2,3 | | P-values  (WNL/CIN1 vs CIN 2,3) | | |
| --- | --- | --- | --- | --- | --- | --- | --- | --- | --- | --- | --- | --- | --- |
|  | 17-29 years | 30-39 years | 40-65 years | P-value (17-29 vs 30-39) | P-value (17-29 vs 40-65) | 17-19 years | 30-39 years | 40-65 years | P-value (17-29 vs 30-39) | P-value (17-29 vs 40-65) | 17-29 years | 30-39 years | 40-65 years |
| # in group | 1510 | 2849 | 3471 | ------------ | ------------ | 36 | 89 | 95 | ------------ | ------------ | ------------ | ------------ | ------------ |
| # of HC2+ | 500 | 503 | 479 | ------------ | ------------ | 34 | 71 | 83 | ------------ | ------------ | ------------ | ------------ | ------------ |
| # HR Types, PCR Done, HC2+ | 403 | 358 | 313 | ------------ | ------------ | 32 | 62 | 71 | ------------ | ------------ | ------------ | ------------ | ------------ |
| **# with HPV 16 (prevalence %)** | 57 (4.7%) | 54 (2.7%) | 58  (2.6%) | 0.0036 | 0.0018 | 8 (23.6%) | 16 (20.6%) | 26 (32.0%) | 0.7253 | 0.3477 | 0.0097 | 0.0001 | < 0.0001 |
| **# with HPV 18** | 48 (3.9%) | 34 (1.7%) | 25 (1.1%) | 0.0003 | < 0.0001 | 8 (23.6%) | 5 (6.4%) | 4 (4.9%) | 0.0277 | 0.0149 | 0.0072 | 0.0887 | 0.1127 |
| # with HPV 31 | 31 (2.5%) | 33 (1.6%) | 25 (1.1%) | 0.0817 | 0.0038 | 8 (23.6%) | 4 (5.2%) | 5 (6.2%) | 0.0166 | 0.0245 | 0.0039 | 0.1625 | 0.0588 |
| # with HPV 33 | 43 (3.5%) | 23 (1.1%) | 16 (0.7%) | < 0.0001 | < 0.0001 | 8 (23.6%) | 9 (11.6%) | 4 (4.9%) | 0.1395 | 0.0149 | 0.0060 | 0.0040 | 0.0795 |
| **# with HPV 35** | 80 (6.6%) | 61 (3.0%) | 51 (2.3%) | <0.0001 | < 0.0001 | 9 (26.6%) | 16 (20.6%) | 18 (22.2%) | 0.4997 | 0.6187 | 0.0087 | 0.0001 | < 0.0001 |
| # with HPV 39 | 33 (2.7%) | 16 (0.8%) | 9  (0.4%) | 0.0001 | < 0.0001 | 2  (5.9%) | 1 (1.3%) | 0  (0%) | 0.2769 | 0.2016 | 0.4335 | 0.7003 | 0.8033 |
| **# with HPV 45** | 44 (3.6%) | 50 (2.5%) | 31 (1.4%) | 0.0680 | 0.0001 | 1  (3.0%) | 5 (6.4%) | 13 (16.0%) | 0.3867 | 0.0090 | 0.8223 | 0.1566 | 0.0003 |
| # with HPV 51 | 47 (3.9%) | 27 (1.3%) | 24 (1.1%) | < 0.0001 | < 0.0001 | 1  (3.0%) | 3 (3.9%) | 2 (2.5%) | 0.8027 | 0.8846 | 0.7583 | 0.2500 | 0.4178 |
| **# with HPV 52** | 36 (3.0%) | 43 (2.1%) | 32 (1.4%) | 0.1464 | 0.0042 | 3  (8.9%) | 7 (9.0%) | 5 (6.2%) | 0.9792 | 0.6270 | 0.2292 | 0.0345 | 0.0763 |
| # with HPV 56 | 35  (2.9%) | 20  (1.0%) | 16  (0.7%) | 0.0003 | < 0.0001 | 0  (0%) | 3 (3.9%) | 1 (1.2%) | 0.4283 | 0.9188 | 0.5101 | 0.1904 | 0.6707 |
| **# with HPV 58** | 53 (4.4%) | 39 (1.9%) | 37 (1.6%) | 0.0002 | < 0.0001 | 7 (20.7%) | 7 (9.0%) | 8 (9.8%) | 0.1287 | 0.1599 | 0.0194 | 0.0296 | 0.0131 |
| # with HPV 59 | 39 (3.2%) | 22 (1.1%) | 18 (0.8%) | 0.0001 | < 0.0001 | 1  (3.0%) | 0  (0%) | 2 (2.5%) | 0.4485 | 0.8846 | 0.9316 | 0.6367 | 0.3346 |
| # with HPV 68 | 55 (4.5%) | 23 (1.1%) | 23 (1.0%) | < 0.0001 | < 0.0001 | 3  (8.9%) | 1 (1.3%) | 2 (2.5%) | 0.1336 | 0.2165 | 0.3778 | 0.9066 | 0.4031 |
